# An NADPH oxidase regulates carbon metabolism and the cell cycle during root nodule symbiosis in common bean (*Phaseolus vulgaris*)

**DOI:** 10.1186/s12870-021-03060-z

**Published:** 2021-06-15

**Authors:** Citlali Fonseca-García, Noreide Nava, Miguel Lara, Carmen Quinto

**Affiliations:** Departamento de Biología Molecular de Plantas, Instituto de Biotecnología, Universidad Nacional Autónoma de México, Avenida Universidad, Cuernavaca, Morelos, Colonia Chamilpa Mexico

**Keywords:** Transcriptome, Common bean, *PvRbohB*, Nodulation, Symbiosis

## Abstract

**Background:**

Rhizobium–legume symbiosis is a specific, coordinated interaction that results in the formation of a root nodule, where biological nitrogen fixation occurs. NADPH oxidases, or Respiratory Burst Oxidase Homologs (RBOHs) in plants, are enzymes that generate superoxide (O_2_^•−^). Superoxide produces other reactive oxygen species (ROS); these ROS regulate different stages of mutualistic interactions. For example, changes in ROS levels are thought to induce ROS scavenging, cell wall remodeling, and changes in phytohormone homeostasis during symbiotic interactions. In common bean (*Phaseolus vulgaris*), *PvRbohB* plays a key role in the early stages of nodulation.

**Results:**

In this study, to explore the role of *PvRbohB* in root nodule symbiosis, we analyzed transcriptomic data from the roots of common bean under control conditions (transgenic roots without construction) and roots with downregulated expression of *PvRbohB* (by RNA interference) non-inoculated and inoculated with *R. tropici*. Our results suggest that ROS produced by PvRBOHB play a central role in infection thread formation and nodule organogenesis through crosstalk with flavonoids, carbon metabolism, cell cycle regulation, and the plant hormones auxin and cytokinin during the early stages of this process.

**Conclusions:**

Our findings provide important insight into the multiple roles of ROS in regulating rhizobia–legume symbiosis.

**Supplementary Information:**

The online version contains supplementary material available at 10.1186/s12870-021-03060-z.

## Background

The mutualistic association between legumes and rhizobia soil bacteria is a specific, symbiotic interaction that results in biological nitrogen fixation. Symbiotic nitrogen fixation occurs in a specialized structure known as the root nodule. The establishment of this interaction requires an exchange of chemical signals between plant roots and the symbiont [1]. Plant roots secrete phenolic compounds (mainly isoflavonoids) into the rhizosphere. These compounds cause the bacterium to synthesize and secrete lipo-chitoligosaccharides called Nodulation (Nod) factors (NFs), which also regulate other rhizobial responses that are crucial for symbiosis, including changes in growth and motility [2, 3]. The rhizobia enter root hair cells at the subapical region, where the plant plasma membrane and cell wall invaginate, to form an infection thread (IT). The IT channels the migration of the rhizobia to dividing cells in the root cortex, where primordial nodule formation occurs. Finally, the bacteria are released into the plant cells that form the nodule and differentiate into bacteroids, which perform biological nitrogen fixation [4]. In legumes, two morphological types of nodules form, depending on where the primordia originate: determinate nodules, which originate from the external cortex, and indeterminate nodules, which originate from the pericycle. Indeterminate nodules have a persistent meristem and are cylindrical, whereas determinate nodules are generally spherical, without a persistent meristem [5].

**Fig. 1:**
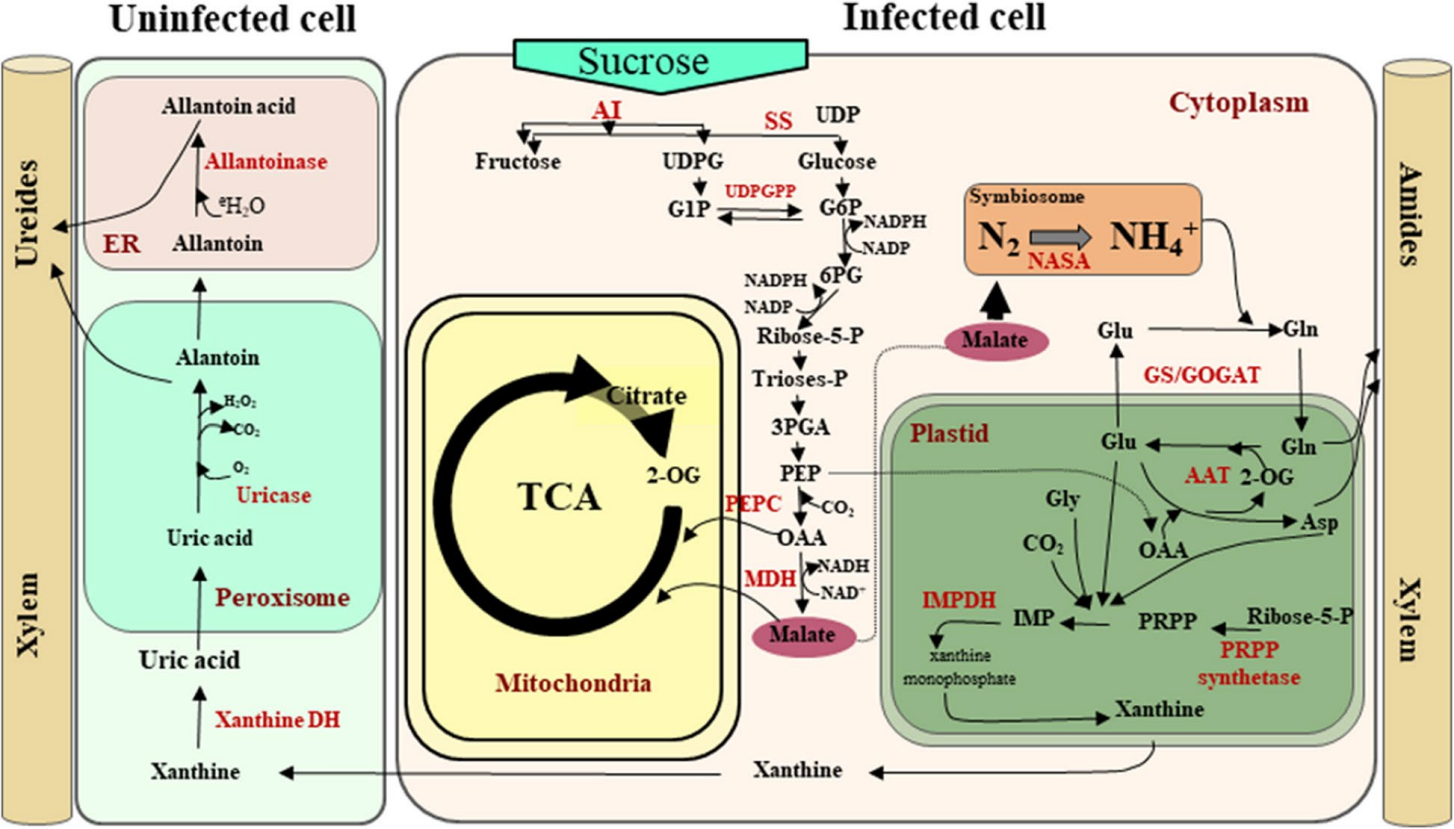
Schematic representation of the major metabolic pathways present in determinate nodules of legumes. The figure illustrates the biosynthesis of nitrogen compounds in infected and uninfected cells, together with sucrose catabolism from the aerial parts of the plant to symbiosomes. NASA: nitrogenase; Glu: glutamate; Gln: glutamine; 2OG: 2-oxoglutarate; OAA: oxaloacetate; Asp: aspartate; GS: glutamine synthetase; GOGAT: glutamate synthase; AAT: aspartate aminotransferase; PRPP: phosphoribosyl pyrophosphate; IMPDH: inosine-5′-monophosphate dehydrogenase; Xanthine DH: xanthine dehydrogenase; UDP: uridine diphosphate; SS: sucrose synthase; AI: alkaline invertase; UDPGTP: uridine diphosphate glucuronosyltransferase; PEPC: phosphoenolpyruvate carboxylase; MDH: malate dehydrogenase

The legume–rhizobia interaction requires coordinated communication between the bacteria and the host plant. This communication allows the rhizobia to be accommodated in the nodule, where atmospheric dinitrogen is reduced into ammonia, providing a source of nitrogen that can be assimilated by the plant. In turn, the plant provides fixed carbon to the rhizobia [4]. In determinate nodules, most of the fixed ammonia is transferred to the plant cytoplasm via diffusion through the bacteroid and symbiosome membranes, where it is rapidly assimilated into glutamine (Gln) by the glutamine synthetase/glutamate synthase (GS/GOGAT) cycle (Fig. 1). Gln is later used as a nitrogen donor for the biosynthesis of amides (Asn and Gln) in indeterminate nodules and purines and ureides in determinate nodules [6–10]. In a similar manner, sucrose from aboveground plant parts is primarily catabolized via sucrose synthase (SS) activity, and the products of this reaction are metabolized through glycolysis to provide carbon skeletons for respiration in the bacteria and ammonium assimilation in the plant [11, 12] (Fig. 1).

Simultaneously, reactive oxygen species (ROS) generated by the NADPH oxidases RBOHs (Respiratory Burst Oxidase Homologs) are detected in specific root tissues at different stages in the legume–rhizobia interaction [13, 14]. In the indeterminate nodules of alfalfa (*Medicago sativa*), superoxide strongly accumulated in the IT and in infected cells in nodules following *Sinorhizobium meliloti* infection [15]. In common bean (*Phaseolus vulgaris*), which forms determinate nodules, we previously detected a clear increase in ROS levels a few seconds after the addition of NFs [16]. This finding was further supported by the observation that transcripts for ROS-generating enzymes such as *PvRbohA*, *PvRbohB*, *PvRbohC*, and *PvRbohD* accumulate during nodule development [17, 18]. In particular, *PvRBOHB* and *PvRBOHA* promoter activity was detected in IT progression sites and at the bases of root hair cells (for *PvRBOHA*) [14, 18]. In a functional analysis in which ROS levels were reduced by RNAi-mediated gene silencing of *PvRbohB* in *P. vulgaris* roots, the advancement of IT was aborted at the base of the epidermal cell [17]. A similar phenotype was observed in *Lotus japonicus* and *Medicago truncatula*, where the IT was severely affected in *LjROP6*-RNAi and *MtROP9*-RNAi roots, resulting in fewer nodules [19, 20]. ROS levels and *MtRbohB* expression were suppressed in *MtROP9*-silenced roots after rhizobial inoculation, pointing to a possible interaction of MtROP9 with RBOH during nodulation [21]. Moreover, overexpressing *PvRbohB* increased ROS production, along with a corresponding increase in IT formation, nodule biomass, and bacteroid number and size in symbiosomes, as well as increased biological fixation of nitrogen [22].

During indeterminate nodule development, superoxide is produced at high levels in pericycle cells [15, 23]. In *M. truncatula*, the *MtRbohE*, *MtRbohF*, and *MtRbohG* promoters were also active in the vascular bundles of nodules [24]. Likewise, in *P. vulgaris*, the promoter activity of *PvRbohB* was observed in vascular bundles of the nodule primordium as well as dividing cortical cells [17]. This observation was confirmed by the finding that RNAi-mediating silencing of *PvRbohB* led to markedly reduced cell division in the outer cortex, suggesting that this gene plays an important role in the developing nodule. Additionally, ROS and phytohormones are needed for rhizobial infection and nodule primordium formation [25–27]. Auxin biosynthesis and signaling occur during IT formation in *M. truncatula* root hairs during the first five days of rhizobial infection [28]. We previously proposed that PvRBOHB plays a positive role in the nodulation of *P. vulgaris* by interacting with diverse signaling mechanisms required for this symbiotic process [17, 22, 29].

We previously obtained an overview of the transcriptome profile of *P. vulgaris PvRbohB*:RNAi roots during nodulation in order to explore the role of *PvRbohB* in this symbiotic interaction; we detected considerable transcriptomic changes during the early stages of root nodule symbiosis with *P. vulgaris* [29]. Furthermore, we identified a collection of differentially expressed genes (DEGs) related to ROS scavenging, cell wall remodeling, and phytohormone homeostasis during nodulation in *P. vulgaris* that were affected by *PvRbohB* silencing. These findings strongly suggest that *PvRbohB* plays a key role in symbiosis by interacting with these metabolic pathways during the early stages of this process.

In the current study, we performed a deeper analysis of the role of *PvRbohB* in nodulation using the same transcriptomic data. We uncovered various molecular mechanisms and metabolic pathways important for root nodule symbiosis. In addition, to confirm the notion that the observed effect of *PvRbohB* silencing was not due to an inactive symbiotic program, we compared the expression profiles of the orthologous genes of *L. japonicus* [30] and *M. truncatula* [31] at the same stage of the nodulation process using available data. Our findings shed light on the crucial roles of *PvRbohB* in legume–rhizobia symbiosis.

## Results

In the present study, we analyzed previously generated transcriptomic data from *PvRbohB*-RNAi roots inoculated with *R. tropici* compared to nonsilenced transgenic roots to elucidate the interaction of PvRBOHB with different metabolic pathways and molecular mechanisms, and thus to better understand the role of this protein in root nodule symbiosis. We also compared our data with other available transcriptomic data from orthologous *L. japonicus* [30, 32] and *M. truncatula* [31, 33] genes at the same stage of the nodulation process.

### The effect of *PvRbohB* on the expression of genes related to the flavonoid biosynthesis pathway under nodulation conditions

Isoflavonoids are secondary metabolites that participate in early signaling and function as chemoattractants. Isoflavonoids also regulate rhizobial responses that are crucial for symbiosis, including growth and motility [2, 3]. In fact, rhizobial colonization increases the production of flavonoids, specifically flavonoids required for the crosstalk of signaling pathways involved in this process [34–38]. *PvRbohB* silencing notably impeded the progression of ITs into inner root cells; the ITs arrested at the base of the root hair. Therefore, we assessed isoflavonoid biosynthesis-related genes, since during this infection process, isoflavonoid biosynthesis increases in response to rhizobia or NFs [39].

**Fig. 2:**
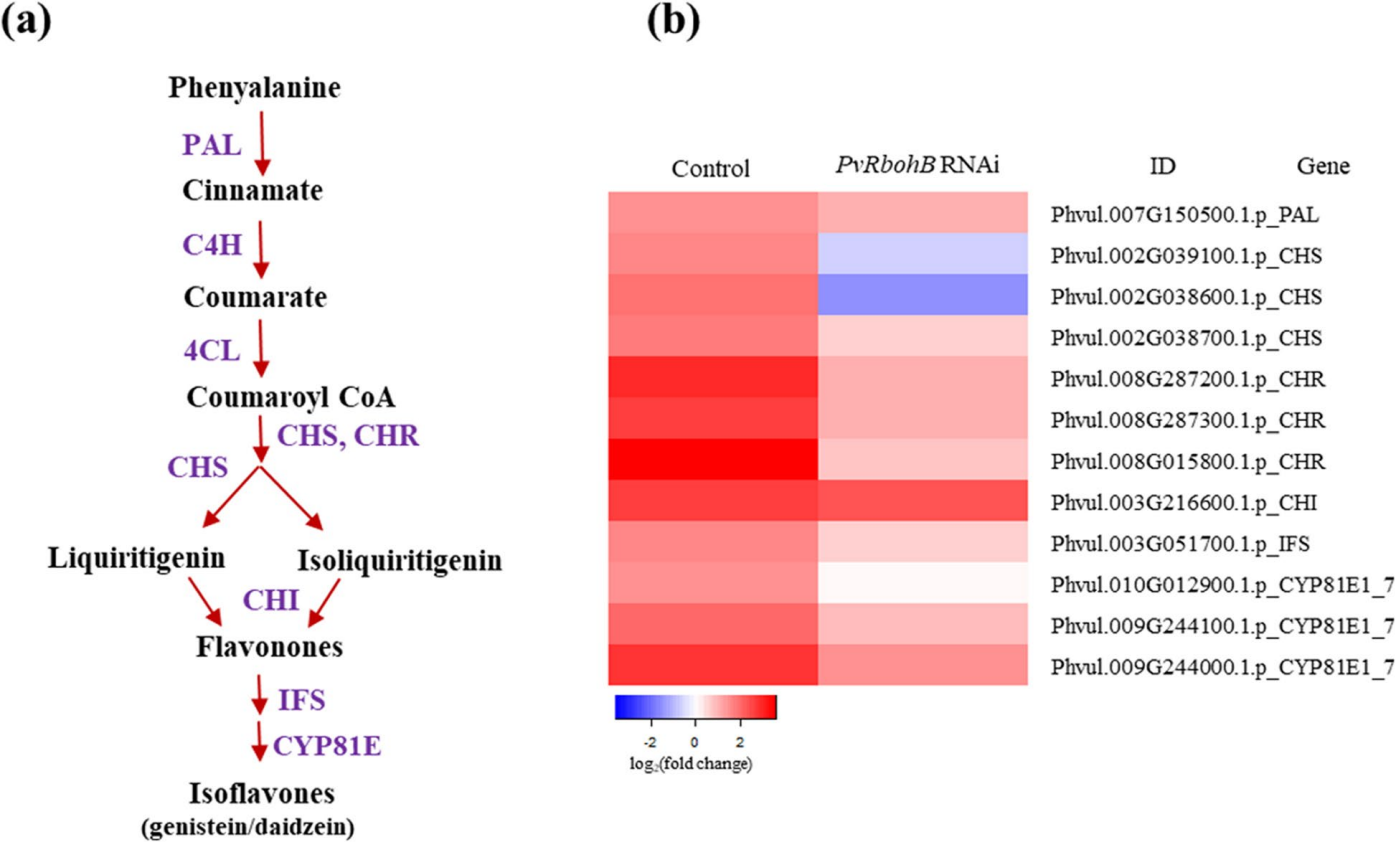
Expression patterns of genes related to the isoflavonoid biosynthesis pathway. **a** Schematic representation of the isoflavonoid biosynthesis pathway in legumes. **b** Differentially expressed genes (DEGs) encoding proteins related to isoflavonoid biosynthesis in control and *PvRbohB*-RNAi *P. vulgaris* roots at 7 dpi with *R. tropici*. The color bars represent the log_2_(fold change), with red representing upregulated genes and blue representing downregulated genes. A cut-off threshold of log_2_FC ≥ 1.5, Padj/FDR ≤ 0.05 was used. PAL: phenylalanine ammonia-lyase; C4H: cinnamate 4-hydroxylase; 4CL: 4-coumarate CoA-ligase; CHS: chalcone synthase; CHR: chalcone reductase; CHI: chalcone isomerase; IFS: isoflavone synthase; CYP81E: isoflavone/4'-methoxyisoflavone 2'-hydroxylase

Isoflavonoids are low molecular weight secondary metabolites containing a 15-carbon skeleton composed of two benzene rings (ring A and ring B) connected by a 3-carbon chain. Isoflavonoids are synthesized through the phenylpropanoid pathway via the activity of chalcone synthase (CHS), the first committed enzyme in flavonoid biosynthesis, producing naringenin chalcone, which is cyclized to naringenin by chalcone isomerase (CHI) (Fig. 2a). In the current study, the expression levels of 12 flavonoid biosynthesis-related genes were higher in control roots inoculated with rhizobia vs. the noninoculated control. Most of these genes encode key enzymes in this pathway, such as phenylalanine ammonia-lyase (PAL), CHS, chalcone reductase (CHR), CHI, and isoflavone synthase (IFS) (Fig. 2b). However, in *PvRbohB*-silenced roots, the expression of these genes was reduced and the levels of the two CHS isoenzymes were markedly downregulated, in contrast to CHI, whose level was not modified (Fig. 2b). These results suggest that *PvRbohB* preferentially regulates isoflavone content rather than the contents of phenolic compounds during symbiosis.

Interestingly, the orthologous genes involved in flavonoid biosynthesis were downregulated in *L. japonicus* and *M. truncatula*, except for genes encoding CHI, which were highly expressed in *L. japonicus* (Additional file 1: Figure S1). These results indicate that isoflavonoid biosynthesis is active in *P. vulgaris*, mostly inactive in *L. japonicus*, and inactive in *M. truncatula* during the early stages of symbiosis. In *PvRbohB*-RNAi roots, the regulation of all steps in this pathway tended to decrease, pointing to a role for the ROS produced by PvRBOHB upon isoflavonoid biosynthesis in *P. vulgaris*. This notion is in agreement with the finding that flavonoid biosynthesis is stimulated by ROS [40] and that ROS are present in the nodule primordia and IT.

### Effects of *PvRbohB* on the assimilation and transport of nitrogen fixed by rhizobia

**Fig. 3:**
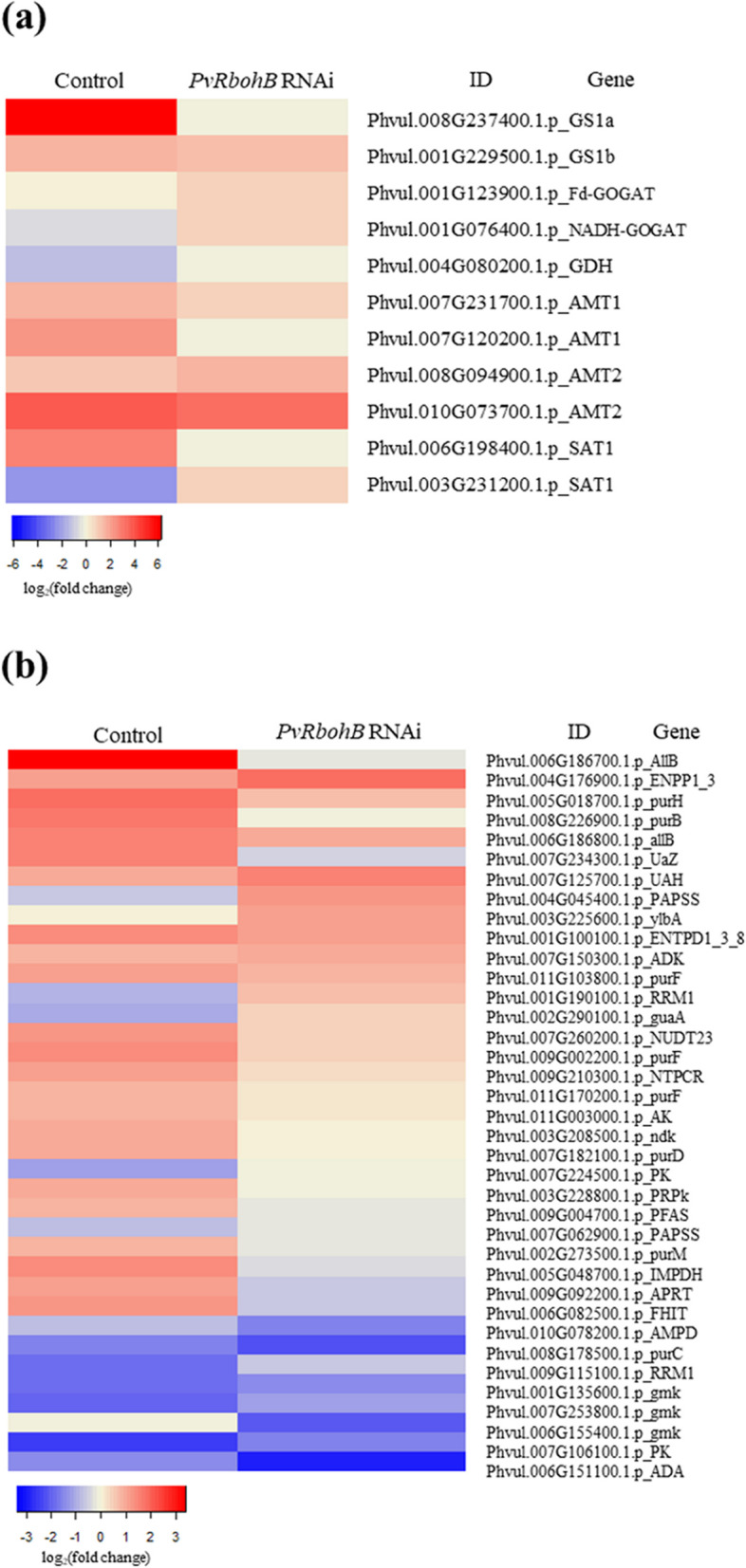
Expression profiles of genes related to the assimilation and transport of nitrogen fixed by rhizobia. **a** DEGs related to fixed ammonia assimilation and transmembrane transporters in nodules and **b** the expression patterns of genes related to ureide biosynthesis in control and *PvRbohB*-RNAi *P. vulgaris* roots at 7 dpi with rhizobia. The color bars represent the log_2_(fold change), with red representing upregulated genes and blue representing downregulated genes. A cut-off threshold of log_2_FC ≥ 1.5, Padj/FDR ≤ 0.05 was used

Based by the finding that the downregulation of *PvRbohB* reduced the amount of nitrogen fixed by rhizobia by 90% [17], we evaluated the transport and assimilation of ammonia in control and *PvRbohB*-silenced roots. Genes involved in the GS1a/GOGAT cycle were upregulated in the determinate nodules of control roots, whereas *GS1b* expression remained nearly constant (Fig. 3a). This result is in agreement with the finding that a nodule-specific GS isoform is induced in common bean, as GS is the main enzyme responsible for the assimilation of ammonia [41]. *GS1a* expression was drastically reduced in *PvRbohB*-silenced plants, which is in accordance with our previous finding that nitrogen fixation is reduced in these plants [17]. Unexpectedly, *GOGAT* gene expression was lower in control vs. *PvRbohB*-silenced plants. As GOGAT catalyzes the deamidation of glutamine to produce glutamic acid, our results indicate that glutamine is preferentially synthesized rather than glutamic acid. This notion is supported by the high demand for glutamine for purine and ureide biosynthesis. In the three legumes analyzed, genes encoding glutamate dehydrogenase (GDH) were similarly repressed, indicating that this enzyme does not participate in ammonia assimilation during symbiosis (Fig. 3a). Most genes encoding transmembrane ammonium transporters (AMT1-2 and SAT1 [Symbiotic Ammonium Transporter 1]) were upregulated in control roots, and their expression was slightly reduced in *PvRbohB*-silenced *P. vulgaris* roots (Fig. 3a, Additional file 1: Figure S2). These results indicate that ammonium is actively transported and assimilated during symbiosis in common bean and that *PvRbohB* fine-tunes nitrogen metabolism in this plant.

Legumes that form determinate nodules generally synthesize ureides to distribute and assimilate fixed nitrogen to the aerial parts of the plant with some exceptions [6–8]. Ureides are derived from de novo synthesized purines that are subsequently oxidized to allantoin and allantoic acid in uninfected cells in the central zone of the nodule. To identify the beginning of this pathway and to explore its regulation during nodule formation, we analyzed the genes involved in purine metabolism. In control roots, we identified 23 upregulated genes and 12 downregulated genes (Fig. 3b). Genes encoding uricase and allantoinase, the responsible enzymes for the production of uric acid and allantoic acid, respectively, showed the most obvious induction. Nevertheless, in *PvRbohB*-RNAi roots, approximately 75% of the genes involved in purine synthesis and those involved in ureide synthesis displayed a notable decrease in expression. However, this expression pattern was not observed in the *L. japonicus* roots, where only a few genes were upregulated (Additional file 1: Figure S3). These results confirm that lotus and beans having a different regulatory mechanism at this stage of nodule symbiosis in these plants, even though beans and lotus both form determinate nodules.

### Effect of *PvRbohB* on the expression of genes related to carbon metabolism in the roots of transgenic bean plants inoculated with rhizobia

The correct balance between carbon and nitrogen metabolism is required for the proper functioning of the nodule, as this balance results in the sufficient supply of nitrogen for plant development [4]. In the current study, we demonstrated that nitrogen metabolism is active prior to the formation of a mature determinate nodule. Nonetheless, in *P. vulgaris* roots inoculated with *R. tropici*, ureide biosynthesis was considerably affected by *PvRbohB* silencing. Therefore, we investigated whether this observed effect on the ureide pathway is also influenced by other factors related to carbon metabolism during early stages of nodule symbiosis. To evaluate this hypothesis, we analyzed the pentose phosphate pathway (PPP), as ribose-5-phosphate is an important purine-ureide precursor (Fig. 1).

**Fig. 4:**
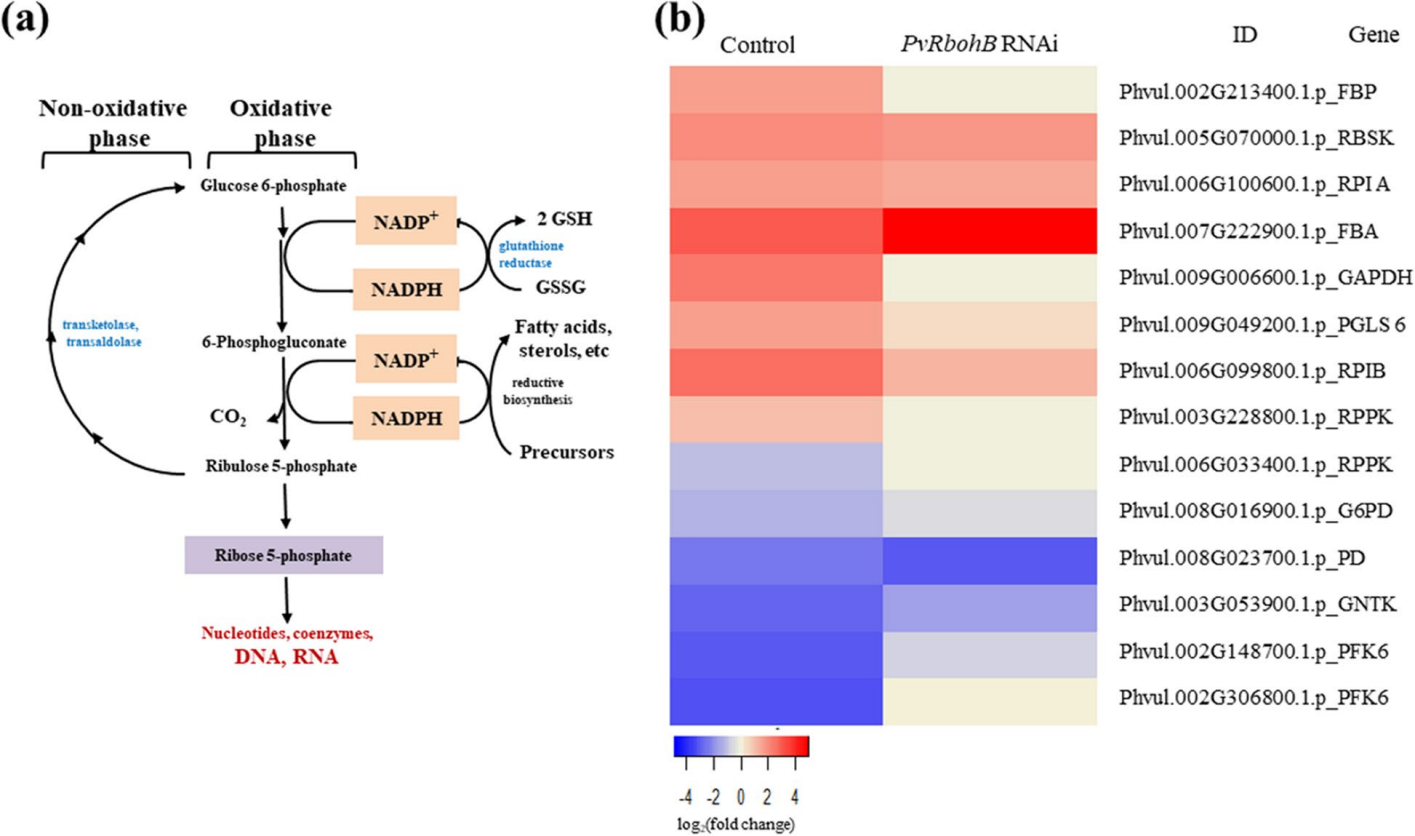
Expression patterns of genes related to carbon metabolism in transgenic roots infected with rhizobia. **a** Schematic representation of the pentose phosphate pathway (PPP). **b** DEGs related to the oxidative and non-oxidative phases of the PPP in control and *PvRbohB*-RNAi *P. vulgaris* roots at 7 dpi with rhizobia. The color bars represent the log_2_(fold change), with red representing upregulated genes and blue representing downregulated genes. A cut-off threshold of log_2_FC ≥ 1.5, Padj/FDR ≤ 0.05 was used

Key genes involved in the oxidative and non-oxidative phases of the PPP were upregulated in *P. vulgaris* control roots at 7 dpi with rhizobia (Fig. 4b), suggesting that this pathway is active along with purine-ureide synthesis in these tissues. Moreover, we detected a drastic reduction in the accumulation of 6-phosphofructokinase 1 (PFK6) in control bean roots, indicating that glucose metabolism through the glycolysis pathway was reduced, supporting the notion that carbon flow increases through the PPP. Conversely, this group of PPP genes showed a low level of expression in *PvRbohB*:RNAi roots in conjunction with increased *PFK6* expression, indicating that PPP activity was clearly reduced and glycolysis increased in these tissues. However, a different expression pattern was observed in *L. japonicus* and *M. truncatula* samples (Additional file 1: Figure S4), where only approximately three genes were upregulated, supporting the notion that a different regulatory mechanism functions at this stage of nodule symbiosis in these model legumes (which are developing determinate and indeterminate nodules, respectively).

During legume–rhizobia symbiosis, sucrose from aboveground plant parts is catabolized, primarily through sucrose synthase activity, and the products of this reaction are metabolized via glycolysis to provide carbon skeletons for bacteroidal respiration and ammonium assimilation [11, 12] (Fig. 1). Since ureide synthesis and the pentose phosphate pathway were affected in *PvRbohB*:RNAi roots, we assessed the expression of genes involved in providing carbon skeletons from sucrose to bacteroids. Interestingly, most genes related to sucrose catabolism were downregulated in control and *PvRbohB*:RNAi roots, with a slight increase in the expression ratio in the latter (Additional file 1: Figure S5). This result is in agreement with the reduced flow of carbon through glycolysis, since *PFK6* was repressed in control plants. These results suggest that carbon skeletons are generated from glyceraldehyde 3-phosphate produced in the PPP and not from that produced via glycolysis. Nonetheless, sucrose catabolism was slightly more active in *L. japonicus* and *M. truncatula* than in *P. vulgaris* (Additional file 1: Figure S5), suggesting that metabolism targeted at supplying carbon skeletons to the microsymbiont is activated starting at the beginning of nodule formation in these two model legumes.

### Effect of *PvRbohB* on the expression of genes related to the cell cycle in root nodules

**Fig. 5:**
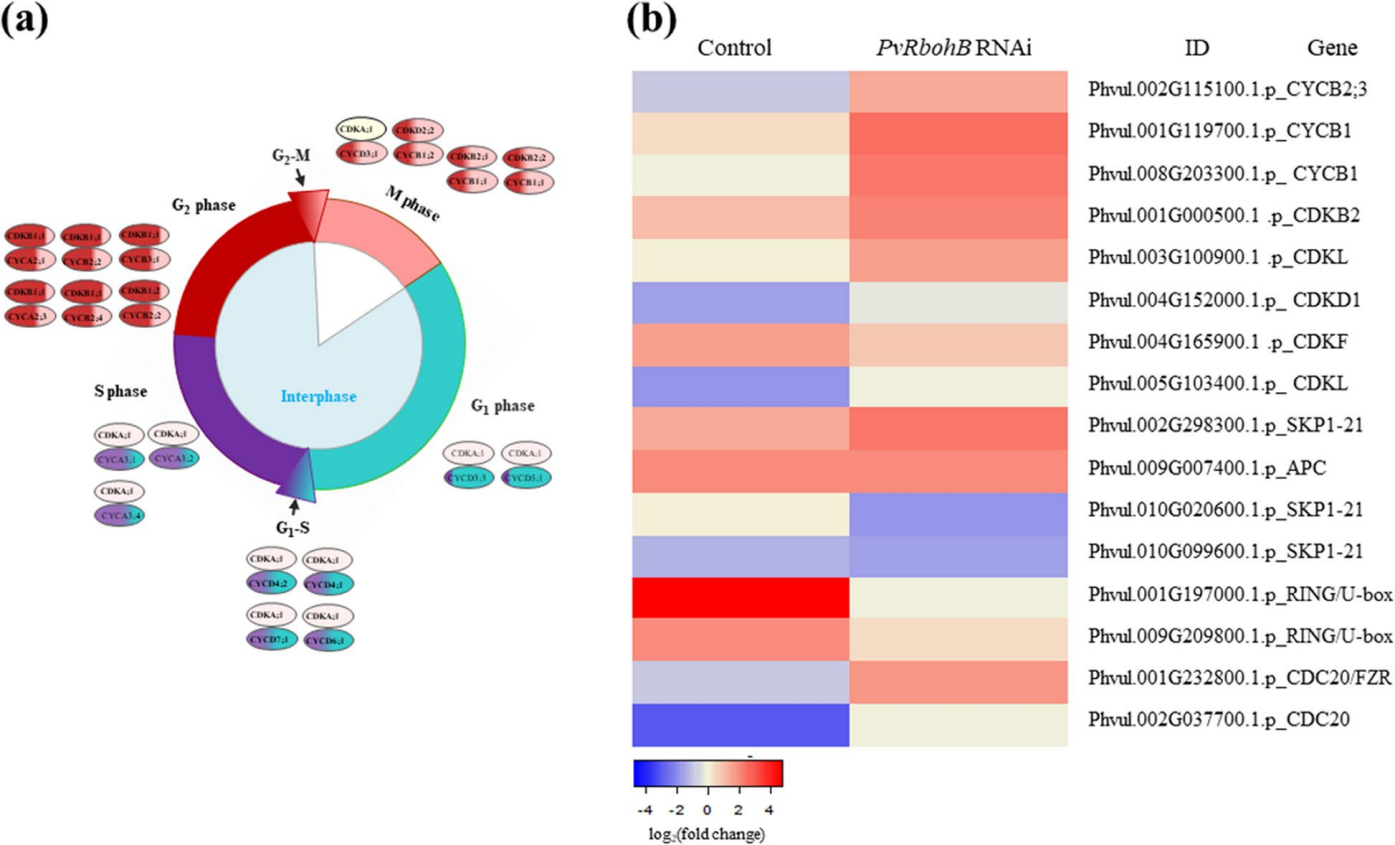
Gene expression profiling of genes that regulate the cell cycle in root nodules. **a** Model of cell cycle control in plants. Different cyclins (CYC) and cyclin-dependent kinases (CDK) are expressed at specific time points of the mitotic cycle, and their combinatorial interactions promote the different phases of the cell cycle. Genes upregulated at the G_1_, S, G_2_, and M phases are labeled in blue, purple, red, and pink, respectively. **b** DEGs involved in regulating the cell cycle in control and *PvRbohB*-RNAi *P. vulgaris* roots at 7 dpi with rhizobia. The color bars represent the log_2_(fold change) of the DEG, with red and blue representing upregulated and downregulated genes, respectively. A cut-off threshold of log_2_FC ≥ 1.5, Padj/FDR ≤ 0.05 was used

Different types of cells and layers must coordinate their development to ensure successful nodule organogenesis. In determinate nodules, the first cell division events typically occur in the outer cortex [5]. Cell proliferation occurs through the mitotic cell cycle driven by the periodic activation of cyclin-dependent kinases (CDKs) which, in combination with different cyclins (CYCs), activate the transition from the G_1_ to S phase and the G_2_ to M phase of the cell cycle (Fig. 5a) [42]. We therefore analyzed the expression of genes involved in the cell cycle in control and *PvRbohB*:RNAi *P. vulgaris* roots.

The activity of CYC–CDK complexes, among others, is controlled by the ubiquitin-mediated degradation of cell cycle proteins; this degradation is important for the timely progression of the cell cycle [42]. Two RING/U-boxes related to Skp-Cullin1-F-Box (SCF) E3 ligase, which along with the anaphase-promoting complex/cyclosome (APC/C), are primarily involved in the transition from M to G1 phase (Fig. 5b). The APC/C is a large complex of 11–13 subunit proteins, including a cullin (Apc2) and RING (Apc11) subunit much like SCF. Anaphase starts when APC/C ubiquitinates the inhibitory chaperone securin. The destruction of securin activates a separase, which then breaks down cohesin, a protein responsible for holding sister chromatids together, releasing them to move towards the cell poles. In this manner, APC/C promotes the metaphase-to-anaphase transition, allowing mitosis to be completed and the cells to enter into the G1 phase [43]. Mitotic exit provides a robust mechanism for maintaining cell identity throughout cell division [44]. Genes encoding these proteins were upregulated in control plants. Similarly, a *CDKF* gene encoding a Cyclin-Dependent Kinase 20 was also upregulated in control plants. This gene encodes a catalytic subunit of the cyclin-dependent kinase complex whose activity is restricted to the G1-S phase of the cell cycle, when cells produce the proteins needed for DNA replication during mitosis.

In *PvRbohB*:RNAi roots, approximately 75% of these genes were differentially expressed compared to control roots (Fig. 5b). The *Cyclin B1*, *B2*, and *CDKB2* genes were markedly upregulated. These genes primarily participate in the G2-M phase, and their expression is tightly controlled and restricted to the late G2 and M phases [45, 46]. When chromosomes are properly aligned during anaphase, rapid degradation of cyclin B1 and B2 by APC/C is required for mitotic exit and the completion of the cell cycle. In the same direction, CDK inactivation is believed to occur due to the proteolysis of B2. Moreover, the ectopic expression of B2 and the expression of nondegradable B1 cyclin interrupt the passage from G2 to M and therefore the exit from mitosis [47, 48]. These results indicate that in control plants, the expression of cell cycle genes favors the exit from the M phase, allowing the cell to start a new division cycle. In *PvRbohB*-silenced roots, the expression of the cell cycle genes affects the advance of anaphase and the exit from mitosis. These results are in agreement with the observation that the nodule meristem starts to develop by 7 dpi.

In contrast to our observations in *P. vulgaris*, in *L. japonicus* roots, no significant change in the expression of these genes was observed, while in *M. truncatula*, *CDKD1* was upregulated (Additional file 1: Figure S6). These results suggest that the cell cycles in *L. japonicus* and *M. truncatula* in symbiosis with rhizobia have a different regulatory mechanism from that in *P. vulgaris*, even though both *L. japonicus* and common bean generate determinate nodules.

### RT-qPCR validation of gene expression profiles

**Fig. 6:**
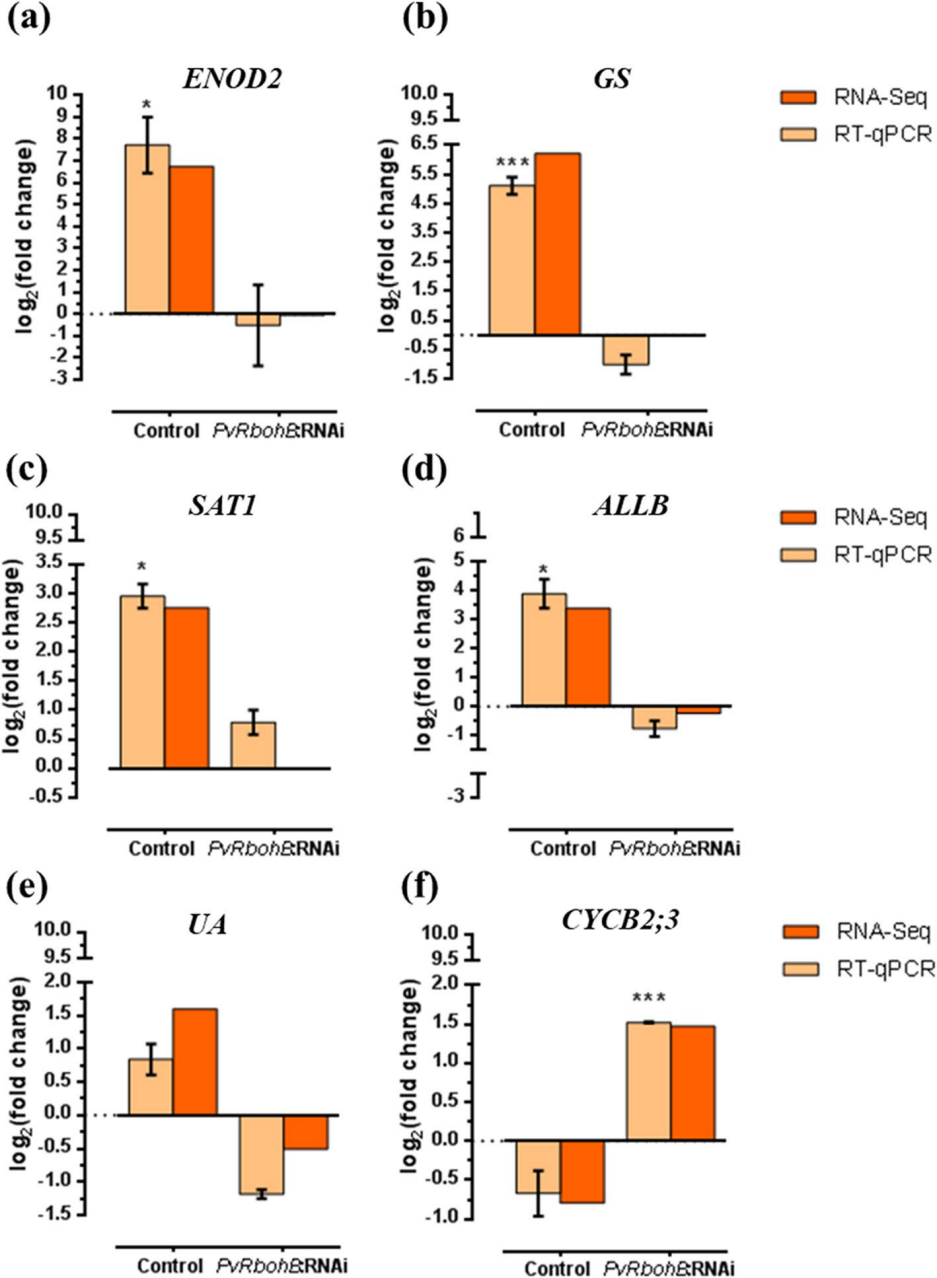
RT-qPCR validation of the gene expression profiles observed in the RNA-Seq data. The transcript levels of *PvENOD2* (**a**), *PvGS* (**b**), *PvSAT1* (**c**), *PvALLB* (**d**), *PvUA* (**e**), and *PvCYCB2;3* (**f**) were measured in control and *PvRbohB*-RNAi transgenic roots at 7 dpi with *R. tropici*. The log_2_(fold change) of the corresponding genes from the RT-qPCR and RNA-Seq data are represented by beige and oranges bars, respectively (**a–f**). The RT-qPCR data were normalized to the expression of *PvEF1α*, and bars show the means ± SE of at least three independent biological replicates, with three technical repeats (*n* > 10). The asterisks represent significant differences between non-inoculated and inoculated samples, as revealed using an unpaired Student’s *t*-test (*P < 0.05; ***P < 0.001)

To validate the results obtained using transcriptome data, we used RT-qPCR to quantify the transcript abundance of six DEGs in control and *PvRbohB*-RNAi roots inoculated with *R. tropici*. The early nodulin gene *EARLY NODULIN 2* (*ENOD2*) was used as a molecular marker of the nodulation process (Fig. 6a). We also evaluated the expression of *CYCB2:3* (involved in the mitotic cell cycle), *GS* and *SAT1* (involved in the assimilation and transport of nitrogen fixed by rhizobia) and *ALL-B* and *UA* (involved in the biosynthesis of ureides to provide the aerial parts of the plant with fixed ammonia). Importantly, in response to inoculation with rhizobia, all of the selected genes showed strong differences in expression, being expressed at high levels in control roots and at low levels in *PvRbohB*-RNAi roots, or vice versa based on RNA-seq. Based on RT-qPCR, *ENOD2* transcript levels were significantly higher in control roots inoculated with rhizobia, but this nodulin marker was not induced in *PvRbohB*-RNAi roots, supporting our previous findings [17, 29]. Moreover, *GS*, *SAT1*, *ALL-B*, and *UA* were upregulated in control roots and downregulated in *PvRbohB*-RNAi roots (Fig. 6b-e). Finally, *CYCB2:3* was upregulated in *PvRbohB*-RNAi roots and downregulated in control roots (Fig. 6f). Therefore, the results obtained using RT-qPCR and RNA-seq were highly consistent, supporting the notion that *PvRbohB* regulates the expression of genes related to the molecular signaling and metabolic pathways evaluated in this study.

## Discussion

Comparative analyses of the metabolic components that participate in rhizobial infection and nodule organogenesis and the regulators of these processes in legume root nodules are crucial for further understanding symbiotic nitrogen fixation. Here, we analyzed previously generated transcriptomic profiling data from control roots and roots with RNAi-induced downregulated *PvRbohB* expression in common bean inoculated with *R. tropici*. The goal of this study was to elucidate the interactions of PvRBOHB with different metabolic pathways in *P. vulgaris* to better understand its role in root nodule symbiosis. For comparison, we used available transcriptomic data from wild-type *L. japonicus* roots [30, 32] and nodules from wild-type *M. truncatula* plants [31, 33].

Legumes and symbiotic soil rhizobia undergo intense molecular signaling [49, 50]. Plant roots excrete flavonoids and isoflavonoids into the rhizosphere [51]. These compounds are perceived by the rhizobia and induce the expression of several genes encoding proteins that synthesize and release NFs, providing signals back the host plant [50, 52]. The NFs increase isoflavonoid biosynthesis in the plant root, thereby creating a positive feedback signal that increases bacterial colonization [53, 54]. Here, we found that isoflavonoid biosynthesis plays an active role in *P. vulgaris* during the early stages (7 dpi) after rhizobial inoculation (Fig. 2). The finding that the downregulation of *PvRbohB* led to decreased levels of CHS isoenzymes, whereas CHI levels were not affected (Fig. 2b), suggests that ROS produced by PvRBOHB affect isoflavone content rather than the contents of phenolic compounds during symbiosis. By contrast, downregulation of flavonoid biosynthesis genes was observed in *L. japonicus* and *M. truncatula *(Additional file 1: Figure 1S). While much attention in the nodulation field has focused on the roles of isoflavonoids, little is known about how their production is regulated during plant–microbe interactions. Our results suggest that isoflavonoid biosynthesis is regulated by ROS. This notion is in accordance with the following findings: flavonoid biosynthesis is induced during the first hours of rhizobial infection until cell division in the inner cortex cells of white clover (*Trifolium repens*) roots [34, 38]; flavonoids are important for nodule initiation in *M. truncatula* [36]; and flavonoids might regulate auxin transport by interacting with peroxidases [36, 38].

In determinate-nodule legumes, fixed atmospheric nitrogen is released as ammonia into the cytoplasm of infected cells [55]. In the cytoplasm, ammonia is assimilated into the amide group of Gln from Glu by the GS-NADH-GOGAT cycle. GS catalyzes the ATP-dependent amidation of Glu to form Gln. GS has been analyzed in various *P. vulgaris* organs [41, 56]. The cytoplasmic GS enzymes are encoded by three distinct genes: *Pvgln-α*, *Pvgln-β*, and the nodule-specific gene *Pvgln-γ*. Promoter analysis of *Pvgln-β* and *Pvgln-γ* in transgenic *Lotus corniculatus* showed that *Pvgln-γ* was preferentially expressed in rhizobia-infected cells of nodules, whereas *Pvgln-β* was expressed at high levels in roots [57]. In the current study, two cytoplasmic *GS* genes (Phvul.008G237400.1.p *PvGS1a* and Phvul.001G229500.1.p *PvGS1b*) were highly expressed during the early stages of nodulation in *P. vulgaris*. Phvul.008G237400.1.p *PvGS1a* showed the highest expression ratio, suggesting that the main GS activity occurs in root nodules at 7 dpi (Fig. 3a). In *M. truncatula* nodules, three *GS* genes have been identified: *MtGS1a* and *MtGS1b* in the cytoplasm and *MtGS2* in the plastid, where *MtGS1a* accounts for 90% of the GS activity in nodules [58, 59].

Additionally, impairing GS activity in *M. truncatula* using phosphinothricin resulted in the inhibition of nodule growth and promotion of nodule senescence [60]. Our data show that Phvul.008G237400.1.p *PvGS1a* expression was notably impaired by the downregulation of *PvRbohB*. Considering that nodule activity in *PvRbohB*:RNAi roots was reduced by 90% compared with the nodules of control roots [17], these results point to the interplay between ROS produced by PvRBOHB and *PvGS* genes in *P. vulgaris* under nodulation conditions. However, Phvul.001G229500.1.p *PvGS1b* remained upregulated in *PvRbohB*-silenced roots, likely due to the weak assimilation of ammonia.

GOGAT catalyzes the transfer of the amide group from glutamine to α-ketoglutarate to yield two glutamate molecules (Fig. 1) [61, 62]. This enzyme is present as two distinct isoforms, NADH-GOGAT and ferredoxin-dependent (Fd) GOGAT. NADH-GOGAT is primarily found in non-green tissues, and its activity and mRNA accumulation increase markedly during nodule development [55, 63, 64]. In the common bean nodule, NADH-GOGAT is present as two isoforms (I and II), with isozyme II showing the most activity during nodule development [64]. However, analysis of a bean root-nodule cDNA library revealed that two distinct cDNAs for NADH-GOGAT were highly expressed in bean nodules [55]. In this study, we found that one *Fd-GOGAT* gene and one *NADH-GOGAT* gene were downregulated in control *P. vulgaris* roots at 7 dpi with rhizobia, but interestingly, both *GOGAT* genes were upregulated in *PvRbohB*:RNAi roots (Fig. 3a). These results indicate that glutamine rather than glutamic acid is preferentially synthesized in control plants, supporting the high demand for glutamine for the synthesis of purines and ureides.

After ammonia is assimilated into glutamine, the amide group of glutamine is used for purine synthesis, producing inosine monophosphate (IMP). IMP dehydrogenase catalyzes the conversion of IMP to xanthine, followed by uric acid (via xanthine dehydrogenase) in infected cells (Fig. 1). Uricase and allantoinase, which have been detected in uninfected cells, catalyze the irreversible conversion of uric acid to allantoin and to allantoic acid, respectively [65]. Allantoin and allantoic acid are the final nitrogen forms that are exported from nodules to the aerial parts of the plant (Fig. 1) [66]. In control *P. vulgaris* roots, several key genes involved in ureide synthesis were highly induced at 7 dpi with rhizobia: Phvul.005G048700.1.p *PvIMPDH*, Phvul.007G234300.1.p *PvUricase*, and Phvul.006G186700.1.p *PvAllantoinase*. By contrast, these crucial genes for ureide biosynthesis were downregulated in *PvRbohB*:RNAi roots. These results demonstrate that even though some transporter genes (*PvAMT2* and *PvSAT1*), *GS*, and *GOGAT* genes were weakly upregulated in these nodules (Fig. 3a), the synthesis of ureides did not maintain the flow of nitrogenous compounds towards the shoots.

In beans, two allantoinase genes *PvALN1* and *PvALN2* while in soybean four genes have been described (*GmALN1* through *GmALN4*). *PvALN1* share greatest sequence similarity with *GmALN1* and *GmALN2* and *PvALN2* was more like *GmALN3* and *GmALN4* [67]. Since *GmALN1* has been reported to be the one with the highest expression in soybean nodules [67], we could suppose that *PvALN1* is the one with the highest expression in bean nodules of control plants (Phvul.006G186700.1p) and its expression is drastically reduced in silenced plants. On the other hand, the allantoinase gene (Phvul.006G186800.1p) could represent the *PvALN2* isoform which shows low expression in both conditions. This result suggests that the common bean *PvALN1* isoform could be responsible for the synthesis of ureides during symbiosis.

Ureide synthesis begins with de novo purine synthesis in the plastids and mitochondria of infected cells [68, 69] (Fig. 1); the nitrogen required for purine synthesis is derived directly from Gln, glycine (Gly), and aspartate (Asp), while the carbon source is contributed by ribose-5-phosphate from the PPP. We observed that the carbon source was also considerably impaired by *PvRbohB* silencing, as genes involved in the oxidative and non-oxidative phases of the PPP showed weaker changes in expression in these nodules than in control roots (Fig. 4b). The central products of the PPP are ribose-5-phosphate and the cofactor NADPH [70, 71], where the latter is used for fatty acid biosynthesis, hydrogen peroxide detoxification, and the maintenance of glutathione in its reduced form by the plant (Fig. 4a). Plants employ RBOHs (NADPH oxidases) to produce ROS in different plant tissues. These enzymes catalyze the reduction of oxygen to generate superoxide anion, using NADPH as an electron donor and generating NADP^+^ [13, 14, 72]. Our results suggest that the downregulation of *PvRbohB* directly affects the PPP reducing the NADP^+^ as an electron donor, thus reducing the availability of the carbon source for the symbionts.

During the early stages of legume–rhizobia interactions, NFs induce cell division in the root cortex, which subsequently develops into the nodule primordium [73]. Determinate nodule formation begins with cell division in the outer cortex. The rapid loss of meristematic activity gives rise to the formation of spherical nodules on tropical legumes such as common bean [5, 73]. Interestingly, the PPP is central for the interconversion of hexoses and pentoses, where ribose 5-phosphate is also precursor for the biosynthesis of nucleotides and nucleic acids, sharing fundamental links with the cell cycle (Fig. 4a). In plants, the re-entry of differentiated cells into the cell cycle often occurs during organ formation or in response to environmental changes [74]. The core cell cycle machinery, including CDK and CYC complexes, is activated during different phases of the cell cycle, thereby regulating these stages in a specific manner (Fig. 5a). At the G1 and S phase, many *CYCD* and *CYCA3* genes are transcribed, and their gene products assembled preferentially with CDKAs. Whereas the CYCD–CDKA complex is preferentially present in cells that transition from the G1 to S phase. At the G2 and M phases, *CYCA2*, *CYCB1*, *CYCB2*, and *CYCD3;1* genes are strongly expressed, and their gene products assemble with CDKB1s, CDKB2s, and CDKA [42].

Here, we observed that the expression profiles of genes likely involved in the transition from the G2 to M phase of the cell cycle in *P. vulgaris* (Phvul.002G115100.1.p *PvCYCB2;3*, Phvul.001G119700.1.p *PvCYCB1*, Phvul.008G203300.1.p *PvCYCB1*, Phvul.001G000500.1.p *PvCDKB2*, and Phvul.004G152000.1.p *PvCDKD1*) were strongly affected by *PvRbohB* silencing (Fig. 5b). Montiel et al. (2012) demonstrated that in *PvRbohB*:RNAi *P. vulgaris* roots inoculated with rhizobia, cell division in the outer cortex was drastically reduced compared to control roots. Additionally, genes involved in the posttranscriptional regulation of the M phase of the cell cycle (Phvul.001G197000.1.p *PvRING/U-box*, Phvul.009G209800.1.p *PvRING/U-box*, Phvul.001G232800.1.p *PvCDC20/FZR*, Phvul.002G037700.1.p *PvCDC20*) showed different expression profiles in *PvRbohB*:RNAi vs. control roots (Fig. 5b).

RING/U-boxes related to SCF E3 ligase, along with anaphase APC/C, promote the transition from the M to G1 phase, allowing mitosis to be completed and the cells to enter the G1 phase. This process allows the cells to be ready to reinitiate cell division and meristem development. By contrast, in *PvRbohB*:RNAi roots, *Cyclin B1*, *B2*, and *CDKB2* gene expression strongly increased. Considering that the protein products of these genes must be degraded to allow mitotic exit and the completion of the cell cycle, these results indicate that in *PvRbohB*:RNAi roots, nodule meristem formation is affected, which is in agreement with our previous results [17]. Together, these results suggest that the ROS produced by PvRBOHB have an important effect on cell cycle regulation at different levels in common bean in symbiosis with rhizobia. Our data are consistent with previous findings about lateral root development, i.e., that molecular and cellular events are regulated by cellular redox status [75–79].

Finally, auxin and cytokinin biosynthesis and signaling are essential for IT formation and nodule organogenesis [26, 27, 80]. In addition, ROS, together with auxin, play a role in cell cycle activation in differentiated leaf cells in alfalfa [74, 81]. We previously reported that genes involved in auxin and cytokinin biosynthesis and transport might interact with ROS produced by PvRBOHB during nodule organogenesis in common bean [17, 29].

## Conclusion

**Fig. 7:**
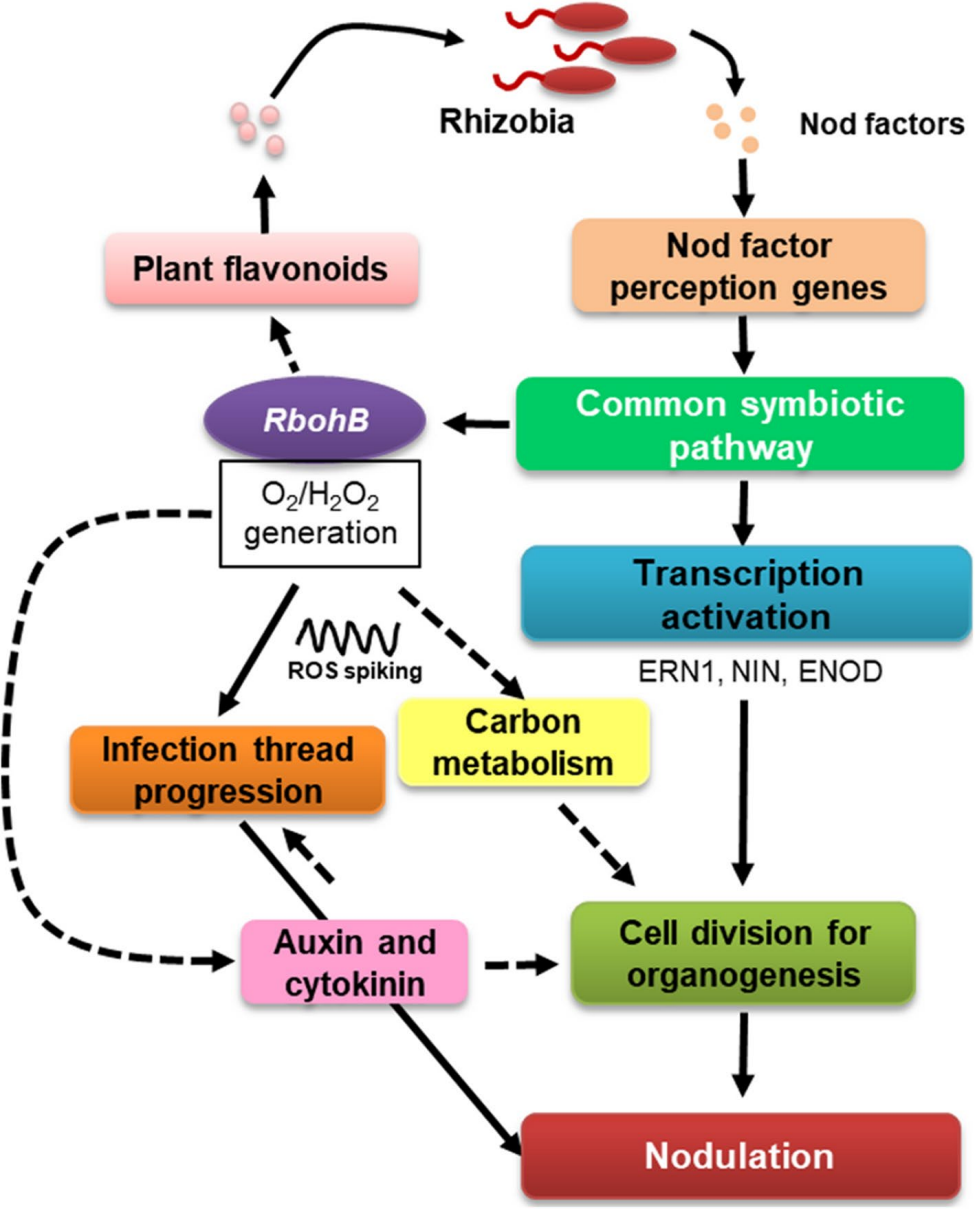
Schematic representation of the role of *PvRbohB* in the early stages of the nodulation process. The model is based on experimental (solid lines) and in silico (dotted lines) data obtained in this study and in previous studies in *P. vulgaris* by our group

In this study, we analyzed transcriptomic data from the roots of common bean under control conditions and roots with downregulated expression of *PvRbohB* non-inoculated and inoculated with *R.* tropici previously performed. Based on the current and previous findings, we propose the following model (Fig. 7): ROS produced by PvRBOHB play a central role in IT and nodule organogenesis via crosstalk with carbon metabolism, cell cycle regulation, and the plant hormones auxin and cytokinin. Functional analysis of molecules with ROS-dependent interactions with these metabolic pathways will increase our understanding of the crosstalk of ROS and rhizobia–legume symbiosis.

## Materials and Methods

### Transcriptome profiling

We previously performed global transcriptome profiling of transgenic *P. vulgaris* cv. Negro Jamapa roots expressing *PvRboh*-RNAi and non-silenced roots inoculated with *R. tropici* or *Rhizophagus irregularis* [29]. In the present study, we employed the same transcriptomic dataset in order to more deeply analyze the role of *PvRbohB* in the rhizobia–common bean interaction. To strengthen our comparison, we included available transcriptomic data from the roots of wild-type *Lotus japonicus* inoculated with *Mesorhizobium loti* at 7 days post inoculation (dpi), respectively, as well *M. truncatula* nodules at 6 dpi with *Sinorhizobium meliloti* [31, 33]. We applied a cutoff threshold of ≥ 1.5 log_2_(fold change) and an FDR-adjusted *P* value of ≤ 0.05 to select DEGs for all transcriptomic data. The distribution and abundance (log_2_ of fold change [FC]) of the DEGs were presented in heat maps and Venn diagrams using the functions heatmap.2 (gplot package) and draw.quad.venn (VennDiagram package), respectively, in the R package. Blast2GO software [82] (https://www.blast2go.com/) was used to the functional annotation of DEGs. An unpaired Student’s *t*-test was performed using the t.test function of the stats package also in R.

### Plant growth conditions and RT-qPCR analysis

In this study, *P. vulgaris* cv. Negro Jamapa was used for RT-qPCR assays. Common bean seeds were obtained from a local market and no specific permits were needed to use. Transgenic *PvRboh*-RNAi and non-silenced (control without construction) roots were generated via transformation using *Agrobacterium rhizogenes* K599 under laboratory conditions according to Instituto de Biotecnología-UNAM guidelines [83]. The composite plants were transferred into pots and inoculated with *R. tropici* strain CIAT899 (1 ml per seedling at OD_600_ = 0.05) under the same conditions used previously [17, 29, 84]. Nodulation was promoted in composite plants that were inoculated with rhizobia, which were irrigated with B&D medium without nitrate (KNO_3_), while the noninoculated plants were watered with B&D solution complemented with 8 mM KNO_3_ to inhibit nodulation. Roots (*n* = 5–10 per condition) were harvested at 7 dpi for RNA isolation, frozen in liquid nitrogen, and stored at –80 °C.

RT-qPCR analysis was performed to identify highly upregulated or downregulated genes in order to validate the RNA-seq data. Total RNA with high-quality was isolated from the stored roots using a ZR Plant RNA MiniPrep kit (Zymo Research) following the manufacturer’s instructions. The RNA integrity was verified by gel electrophoresis, and the RNA concentration was quantified with a NanoDrop2000 spectrophotometer (Thermo Fisher Scientific, Waltham, MA, USA). Genomic DNA was removed using RNase-free DNase (10 U/µl; Roche, Basel, Switzerland) at 37 °C for 30 min. Complementary DNA (cDNA) was synthetized with Thermo Scientific RevertAid Reverse Transcriptase (200 U/µl) using 200 ng of DNA-free RNA as a template following the manufacturer’s instructions. qPCR analysis was performed using a Maxima SYBR Green/ROX qPCR kit (Thermo Fisher Scientific) as follows: each reaction contained 5 μl Maxima SYBR Green/ROX qPCR Master Mix (2X) (Thermo Scientific, USA), 1 μl cDNA sample (20 ng RNA as a template), and 0.33 μl of each primer (10 μM) in a reaction system of 10 μl. The thermal cycling conditions were as follows: 95 °C for 10 min, 40 cycles of 95 °C for 15 s and 60 °C for 60 s. The melting curve stage was evaluated with the following thermal conditions: 95 °C for 15 s, 60 °C for 60 s, and 96 °C for 5 s. A control sample without reverse transcriptase was included in order to corroborate the absence of contaminating DNA. The relative expression values were calculated using the formula 2^–CT^, where the cycle threshold value ΔCt is equivalent to the Ct of the gene of interest minus the Ct of the reference gene (*PvEF1α*) [85]. Three biological replicates with three technical repeats were performed. The list of gene-specific oligonucleotides used for RT-qPCR assay is reported in the supplementary materials Additional file 2: Table S1.

## Supplementary Information

**Additional file 1: Figure S1**. Expression profiles of genes related to the isoflavonoid biosynthesis pathway in legumes. DEGs encoding proteins related to isoflavonoid biosynthesis in control and *PvRbohB*-RNAi*P. vulgaris* roots at 7 dpi with *R. tropici*, together with their orthologous genes from wild-type *L. japonicus* roots inoculated with *M. loti *at 7, as well as *M. truncatula *nodules at 6 dpi with *S. meliloti*. The color bars represent the log_2_(fold change), with red representing upregulated genes and blue representing downregulated genes. A cut-off threshold of log_2_FC ≥ 1.5, Padj/FDR ≤ 0.05 was used. **Figure S2**. DEGs related to fixed ammonia assimilation and transmembrane transporters in nodules. The heatmap shows the DEGs from control and *PvRbohB*-RNAi *P. vulgaris*roots at 7 dpi with *R. tropici*, together with their orthologous genes from wild-type*L. japonicus* roots inoculated with *M. loti *at 7 dpi, as well as *M. truncatula *nodules at 6 dpi with *S. meliloti*. The color bars represent the log_2_(fold change), with red representing upregulated genes and blue representing downregulated genes. A cut-off threshold of log_2_FC ≥ 1.5, Padj/FDR ≤ 0.05 was used. **Figure S3**. Heatmap of the expression patterns of genes related to ureide biosynthesis. DEGs encoding proteins related to ureide metabolism in control and *PvRbohB*-RNAi *P. vulgaris* roots at 7 dpi with rhizobia. Their respective orthologous genes from wild-type, *L. japonicus* roots inoculated with *M. loti *at 7dpi were included in the analysis, along with *M. truncatula *nodules at 6 dpi with *S. meliloti*. The color bars represent the log_2_(fold change), with red representing upregulated genes and blue representing downregulated genes. A cut-off threshold of log_2_FC ≥ 1.5, Padj/FDR ≤ 0.05 was used. **Figure S4**. DEGs related to the oxidative and non-oxidative phases of the Pentose Phosphate Pathway (PPP). Data were analyzed in control and *PvRbohB*-RNAi *P. vulgaris* roots at 7 dpi with rhizobia. Their respective orthologous genes from wild-type *L. japonicus* roots inoculated with *M. loti *at 7 dpi were included in the analysis, along with *M. truncatula *nodules at 6 dpi with *S. meliloti*. The color bars represent the log_2_(fold change), with red representing upregulated genes and blue representing downregulated genes. A cut-off threshold of log_2_FC ≥ 1.5, Padj/FDR ≤ 0.05 was used. **Figure S5**. Expression profiles of genes related to sucrose catabolism to provide carbon skeletons to nodules. DEGs related to sucrose catabolism in control and *PvRbohB*-RNAi *P. vulgaris* roots at 7 dpi with rhizobia; their orthologous genes from wild-type*L. japonicus* roots inoculated with *M. loti *at 7were including in the analysis, along with *M. truncatula *nodules at 6 dpi with *S. meliloti*. The color bars represent the log_2_(fold change), with red representing upregulated genes and blue representing downregulated genes. A cut-off threshold of log_2_FC ≥ 1.5, Padj/FDR ≤ 0.05 was used. **Figure S6**. DEGs involved in regulating the cell cycle in control and *PvRbohB*-RNAi *P. vulgaris* roots. Their respective orthologous genes from wild-type roots of *L. japonicus* inoculated with *M. loti *at 7 dpi were included in the analysis, along with *M. truncatula *nodules at 6 dpi with *S. meliloti* The color bars represent the log_2_(fold change) of the DEG, with red and blue representing upregulated and downregulated genes, respectively. A cut-off threshold of log_2_FC ≥ 1.5, Padj/FDR ≤ 0.05 was used.

**Additional file 2: Table S1.** Gene-specific oligonucleotides used for RT-qPCR analysis.

## Data Availability

The data were retrieved from the NCBI databases under the BioProject accession number PRJNA482464 and the Sequence Read Archive accession numbers SRR7693915–SRR7693917, SRR7696192–SRR7696194, SRR7696200–SRR7696202, SRR7696204–SRR7696206, SRR7696589–SRR7696591, and SRR7696208–SRR7696210.

## Abbreviations

ROS: Reactive oxygen species
RBOH: Respiratory burst oxidase homologs
IT: Infection thread
RNAi: RNA interference
DEG: Differential expressed genes
EF: Elongation Factor
RT-qPCR: Reverse-transcription quantitative of polymerase chain reaction
FDR: False discovery rate
