# Supplementary material for: An NADPH oxidase regulates carbon metabolism and the cell cycle during root nodule symbiosis in common bean (Phaseolus vulgaris)
Source: BMC Plant Biol. 2021 Jun 15;21:274. doi: 10.1186/s12870-021-03060-z (PMC8207584; doi:10.1186/s12870-021-03060-z)
Supplement: Supplementary file 1 — Additional file 1: Figure S1. Expression profiles of genes related to the isoflavonoid biosynthesis pathway in legumes. DEGs encoding proteins related to isoflavonoid biosynthesis in control and PvRbohB-RNAiP. vulgaris roots at 7 dpi with R. tropici, together with their orthologous genes from wild-type L. japonicus roots inoculated with M. loti at 7, as well as M. truncatula nodules at 6 dpi with S. meliloti. The color bars represent the log2(fold change), with red representing upregulated genes and blue representing downregulated genes. A cut-off threshold of log2FC ≥ 1.5, Padj/FDR ≤ 0.05 was used. Figure S2. DEGs related to fixed ammonia assimilation and transmembrane transporters in nodules. The heatmap shows the DEGs from control and PvRbohB-RNAi P. vulgarisroots at 7 dpi with R. tropici, together with their orthologous genes from wild-typeL. japonicus roots inoculated with M. loti at 7 dpi, as well as M. truncatula nodules at 6 dpi with S. meliloti. The color bars represent the log2(fold change), with red representing upregulated genes and blue representing downregulated genes. A cut-off threshold of log2FC ≥ 1.5, Padj/FDR ≤ 0.05 was used. Figure S3. Heatmap of the expression patterns of genes related to ureide biosynthesis. DEGs encoding proteins related to ureide metabolism in control and PvRbohB-RNAi P. vulgaris roots at 7 dpi with rhizobia. Their respective orthologous genes from wild-type, L. japonicus roots inoculated with M. loti at 7dpi were included in the analysis, along with M. truncatula nodules at 6 dpi with S. meliloti. The color bars represent the log2(fold change), with red representing upregulated genes and blue representing downregulated genes. A cut-off threshold of log2FC ≥ 1.5, Padj/FDR ≤ 0.05 was used. Figure S4. DEGs related to the oxidative and non-oxidative phases of the Pentose Phosphate Pathway (PPP). Data were analyzed in control and PvRbohB-RNAi P. vulgaris roots at 7 dpi with rhizobia. Their respective orthologous genes from wild-t [file 12870_2021_3060_MOESM1_ESM.pdf]

Supplementary materials

An NADPH oxidase regulates carbon metabolism and the cell cycle during root nodule symbiosis in common bean (*Phaseolus vulgaris*)

Citlali Fonseca-García, Noreide Nava, Miguel Lara, and Carmen Quinto*

Departamento de Biología Molecular de Plantas, Instituto de Biotecnología, Universidad Nacional Autónoma de México, Avenida Universidad 2001, Colonia Chamilpa, Cuernavaca, Morelos 62210, Mexico.

Citlali Fonseca-García: citlali.fonseca@ibt.unam.mx;

Noreide Nava: noreide.nava@ibt.unam.mx

Miguel Lara: mlara@unam.mx

Carmen Quinto*: carmen.quinto@ibt.unam.mx; Tel.: +52-777-329-1642

*Corresponding author

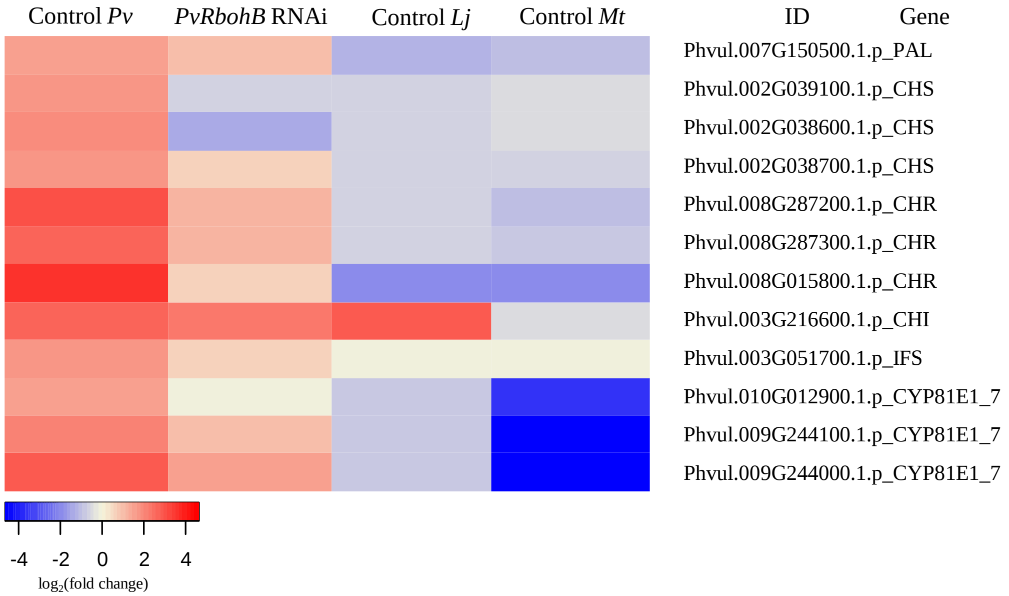

Figure S1. Expression profiles of genes related to the isoflavonoid biosynthesis pathway in legumes. DEGs encoding proteins related to isoflavonoid biosynthesis in control and *PvRbohB*-RNAi *P. vulgaris* roots at 7 dpi with *R. tropici*, together with their orthologous genes from wild-type *L. japonicus* roots inoculated with *M. loti* at 7 dpi, as well as *M. truncatula* nodules at 6 dpi with *S. meliloti*. The color bars represent the log_2(fold change), with red representing upregulated genes and blue representing downregulated genes. A cut-off threshold of $log_2FC \geq 1.5$, Padj/FDR ≤ 0.05 was used.

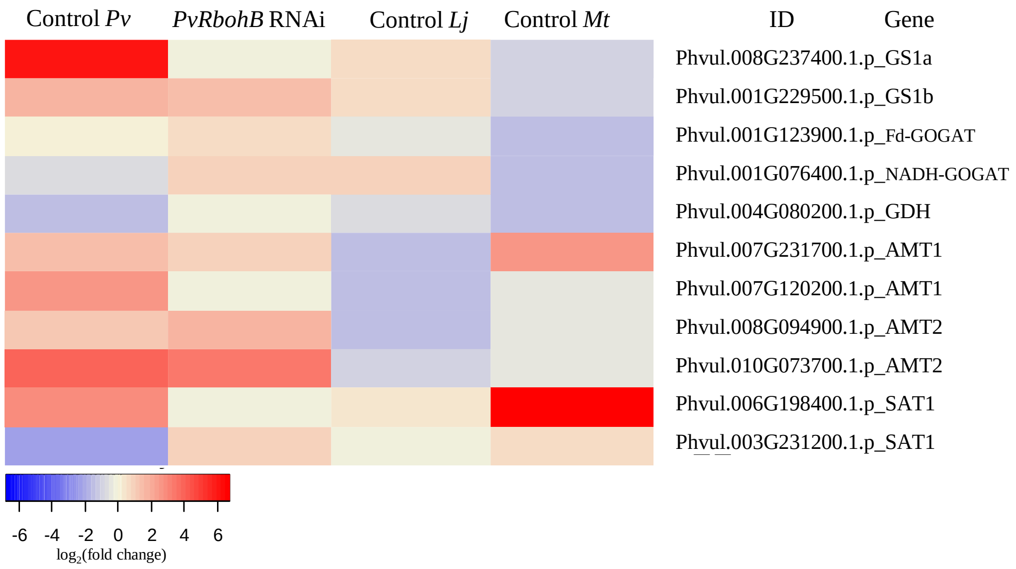

Figure S2. DEGs related to fixed ammonia assimilation and transmembrane transporters in nodules. The heatmap shows the DEGs from control and *PvRbohB*-RNAi *P. vulgaris* roots at 7 dpi with *R. tropici*, together with their orthologous genes from wild-type *L. japonicus* roots inoculated with *M. loti* at 7 dpi, as well as *M. truncatula* nodules at 6 dpi with *S. meliloti*. The color bars represent the $\log_2$(fold change), with red representing upregulated genes and blue representing downregulated genes. A cut-off threshold of $\log_2 FC \geq 1.5$, Padj/FDR ≤ 0.05 was used.

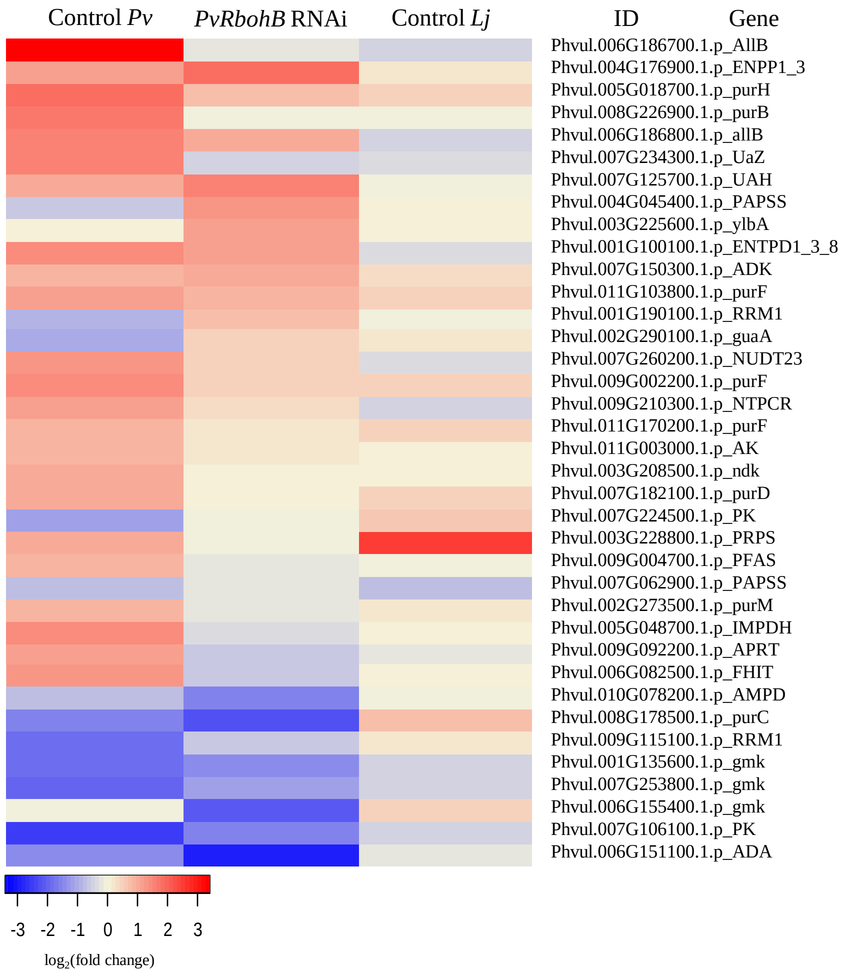

Figure S3. Heatmap of the expression patterns of genes related to ureide biosynthesis. DEGs encoding proteins related to ureide metabolism in control and *PvRbohB*-RNAi *P. vulgaris* roots at 7 dpi with rhizobia. Their respective orthologous genes from wild-type *L. japonicus* roots inoculated with *M. loti* at 7 dpi, were included in the analysis, along with *M. truncatula* nodules at 6 dpi with *S. meliloti*. The color bars represent the $\log_2$(fold change), with red representing upregulated genes and blue representing downregulated genes. A cut-off threshold of $\log_2FC \geq 1.5$, Padj/FDR ≤ 0.05 was used.

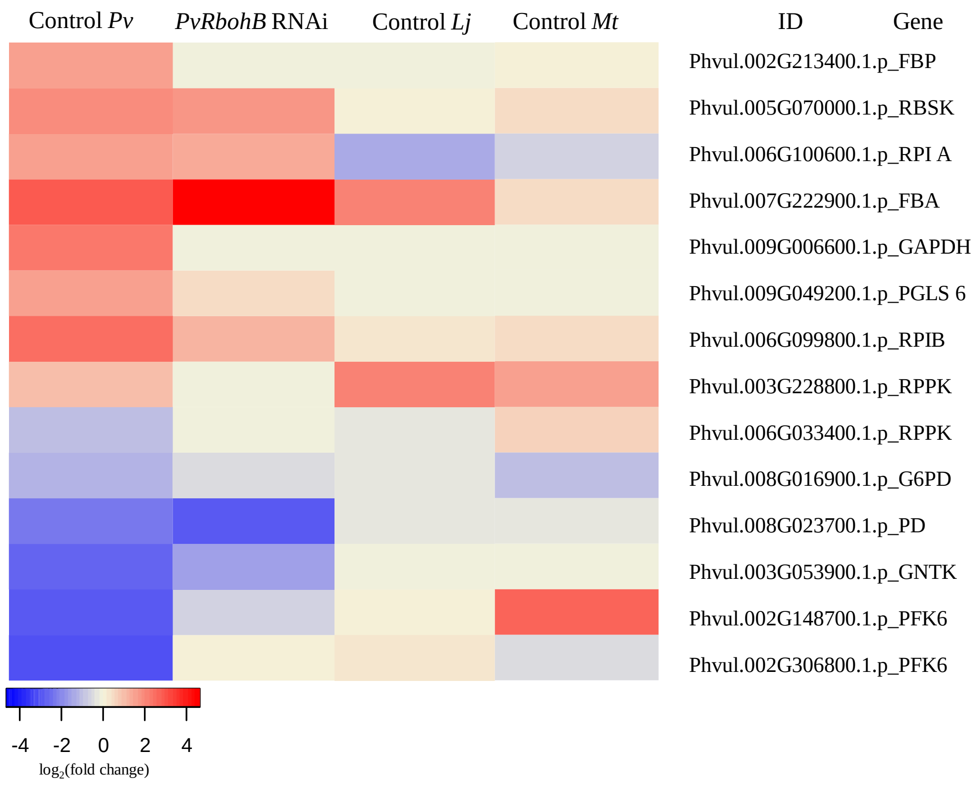

Figure S4. DEGs related to the oxidative and non-oxidative phases of the Pentose Phosphate Pathway (PPP). Data were analyzed in control and *PvRbohB*-RNAi *P. vulgaris* roots at 7 dpi with rhizobia. Their respective orthologous genes from wild-type *L. japonicus* roots inoculated with *M. loti* at 7 dpi were included in the analysis, along with *M. truncatula* nodules at 6 dpi with *S. meliloti*. The color bars represent the $\log_2$(fold change), with red representing upregulated genes and blue representing downregulated genes. A cut-off threshold of $\log_2 FC \geq 1.5$, Padj/FDR ≤ 0.05 was used.

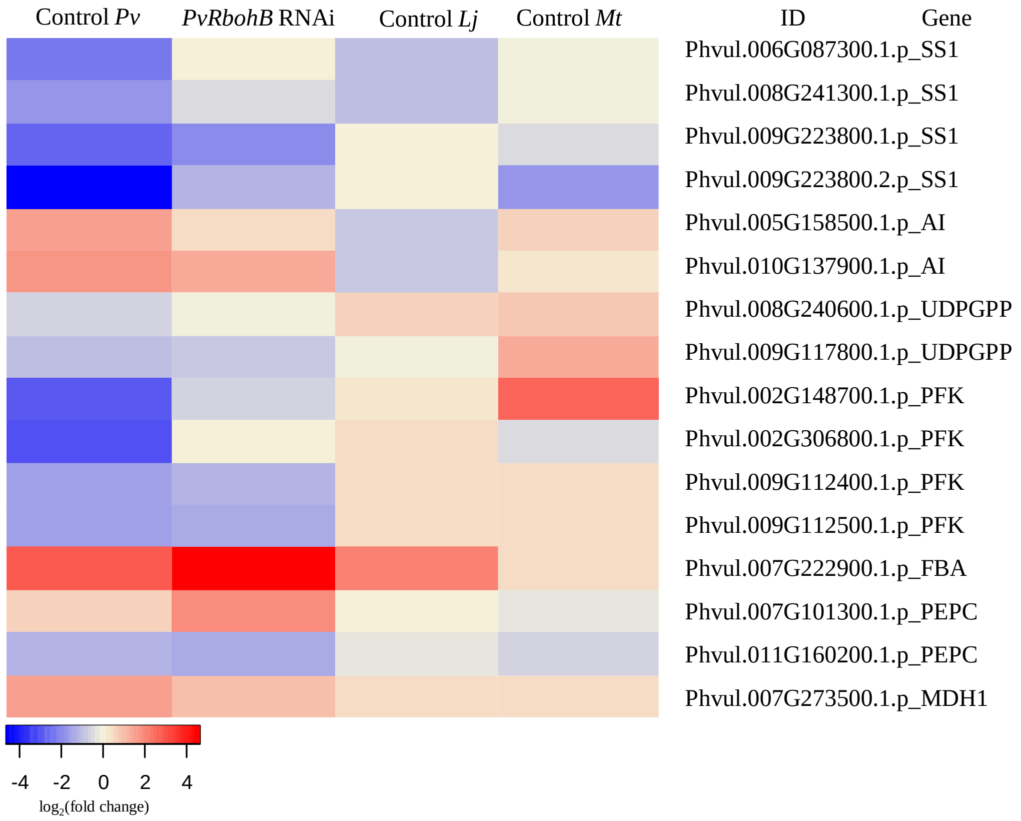

Figure S5. Expression profiles of genes related to sucrose catabolism to provide carbon skeletons to nodules. DEGs related to sucrose catabolism in control and *PvRbohB*-RNAi *P. vulgaris* roots at 7 dpi with rhizobia; their orthologous genes from wild-type *L. japonicus* roots inoculated with *M. loti* at 7 dpi were including in the analysis, along with *M. truncatula* nodules at 6 dpi with *S. meliloti*. The color bars represent the $\log_2$(fold change), with red representing upregulated genes and blue representing downregulated genes. A cut-off threshold of $\log_2 FC \geq 1.5$, Padj/FDR ≤ 0.05 was used.

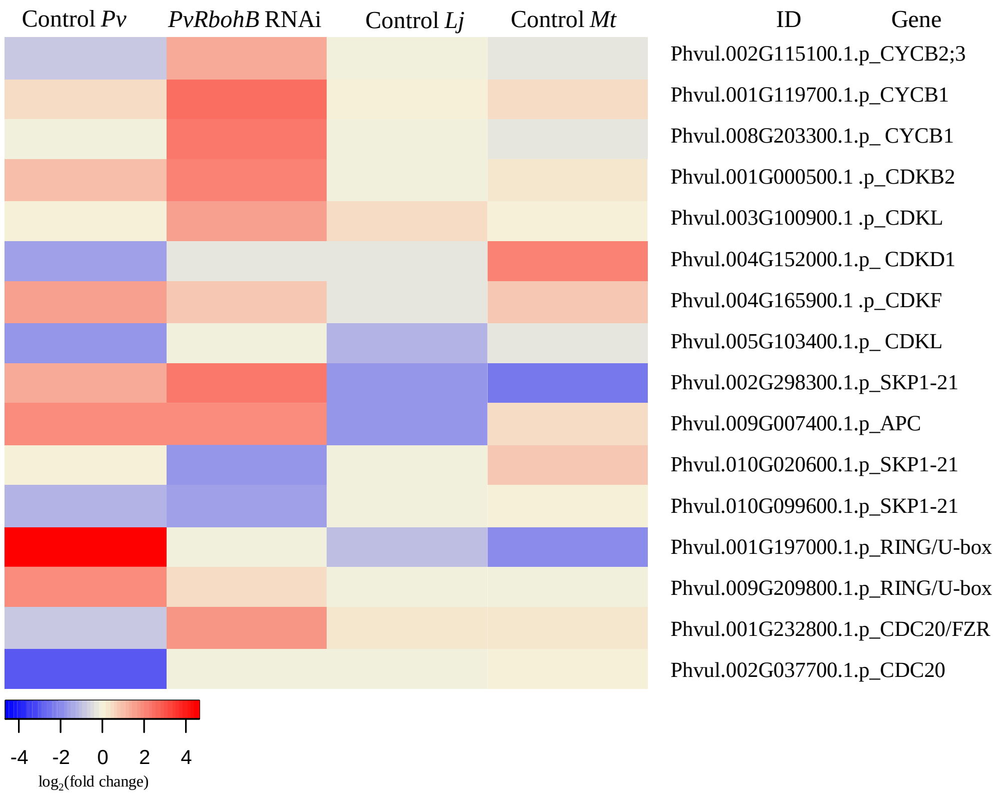

Figure S6. DEGs involved in regulating the cell cycle in control and *PvRbohB*-RNAi *P. vulgaris* roots. Their respective orthologous genes from wild-type roots of *L. japonicus* inoculated with *M. loti* at 7 dpi were included in the analysis, along with *M. truncatula* nodules at 6 dpi with *S. meliloti* The color bars represent the $\log_2$(fold change) of the DEG, with red and blue representing upregulated and downregulated genes, respectively. A cut-off threshold of $\log_2 FC \geq 1.5$, Padj/FDR ≤ 0.05 was used.
