# Supplementary material for: An NADPH oxidase regulates carbon metabolism and the cell cycle during root nodule symbiosis in common bean (Phaseolus vulgaris)
Source: BMC Plant Biol. 2021 Jun 15;21:274. doi: 10.1186/s12870-021-03060-z (PMC8207584; doi:10.1186/s12870-021-03060-z)
Supplement: Supplementary file 2 — Additional file 2: Table S1. Gene-specific oligonucleotides used for RT-qPCR analysis. [file 12870_2021_3060_MOESM2_ESM.pdf]

Table S1. Gene-specific oligonucleotides used for RT-qPCR analysis.

Gene	Tag	Primer sequence (5′→3′)	Amplicon size (bp)	Efficiency (%)
PvEf1α	Ef1α-Up	GGT CAT TGG TCA TGT CGA CTC TGG	146	98
	Ef1α-Lw	GCA CCC AGG CAT ACT TGA ATG ACC		
PvENOD2	ENOD2-Up	AGT GTA CAC ACC CCC ACC ATA CCA	137	ND
	ENOD2-Lw	TCT TGG ATG GTG GAT AGT GGC CA		
PvCyclinB1	CCN-B_Fw	GGC CAA CGC ACA AGA GAA GAA C	137	96
	CCN-B_Rv	CCT CCG ATT CAT CAT CAT CGC		
PvGS	GS_Fw	CAG GAG GGG TAA CAA TAT CCT GG	189	114
	GS_Rv	CAT CCA ACA GGC CAC TGA ACA TC		
PvSAT1	SAT1_Fw	GGG ACT AGG GAA ACC TAA GGT GG	119	91
	SAT1_Rv	GGC TGG CCT TGA AGT GGT AG		
PvALL	ALL_Fw	GTG CTC TCC TCC CAT TCG TG	115	119
	ALL_Rv	CTT GAG TTG AGG CAG GGT TGG		
PvUA	UA_Fw	GCT ATA CAA CCT CCC TCA GAA GCC	111	116
	UA_RvCp	GGG CTA TAG ACT CCC CTG TTT GG		

ND: Not determined
